# Supplementary material for: The need for high-resolution gut microbiome characterization to design efficient strategies for sustainable aquaculture production
Source: Commun Biol. 2024 Oct 25;7:1391. doi: 10.1038/s42003-024-07087-4 (PMC11511968; doi:10.1038/s42003-024-07087-4)
Supplement: Supplementary file 1 — Supplementary Materials [file 42003_2024_7087_MOESM1_ESM.pdf]

Supplementary Information for

The need for high-resolution gut microbiome characterization to design efficient strategies for sustainable aquaculture production

Shashank Gupta[1], Arturo Vera-Ponce de León[1,2], Miyako Kodama[3], Matthias Hoetzinger[1,4,] Cecilie G. Clausen[3], Louisa Pless[3], Ana R.A. Verissimo[3], Bruno Stengel[5], Virginia Calabuig[5], Renate Kvingedal[6], Stanko Skugor[6], Bjørge Westereng[1], Thomas Nelson Harvey[2], Anna Nordborg[7], Stefan Bertilsson[4], Morten T. Limborg[3], Turid Mørkøre[2], Simen R. Sandve[2], Phillip B. Pope[1,2,8], *Torgeir R. Hvidsten[1] and *Sabina Leanti La Rosa[1,2]

[1]Faculty of Chemistry, Biotechnology and Food Science, Norwegian University of Life Sciences, Ås, Norway

[2]Faculty of Biosciences, Norwegian University of Life Sciences, Ås, Norway

[3]Center for Evolutionary Hologenomics, The Globe Institute, University of Copenhagen, Copenhagen, Denmark

[4]Department of Aquatic Sciences and Assessment, Swedish University of Agricultural Sciences, Uppsala, Sweden

[5]Cargill Food Solutions – R&D – SST, Norway

[6]Cargill Aqua Nutrition, Cargill, Sandnes, Norway

[7]Department of Biotechnology and Nanomedicine, SINTEF, Trondheim, Norway

[8]Centre for Microbiome Research, School of Biomedical Sciences, Queensland University of Technology (QUT), Translational Research Institute, Woolloongabba, Queensland, Australia

*These authors contributed equally

Correspondence to: Sabina Leanti La Rosa: sabina.leantilarosa@nmbu.no; Torgeir R. Hvidsten: torgeir.r.hvidsten@nmbu.no

Table of Contents

1. Supplementary results 3
1.1 Growth, biometrics and performance 3
1.2 Feed-associated microbiota 3
1.3 Low mannan inclusion rates have little effect on gut microbial community structure and function 4
1.4 Low mannan inclusion rates have little effect on salmon gene regulation and physiology 7
1.5 A high inclusion β-mannan diet affects the composition of the gut microbiota but has limited effects on salmon metabolism 8
1.6 A high inclusion β-mannan diet has limited effects on salmon metabolism 9
1.7 A high inclusion β-mannan diet has limited impact on the functions of the salmon gut microbiome 10
1.8 A high inclusion β-mannan diet has limited effects on gut SCFAs 11
Supplementary figures 12

1. Supplementary results

1.1 Growth, biometrics and performance

We employed a phenotypic scoring analysis to evaluate the impact of various diets on factors such as weight, length, gutted weight, condition factor, hepatosomatic index, cardio somatic index, welfare indicators and organ integrity for fish fed low dose mannans (**Supplementary Data 4c)**. There were no significant differences in any of the phenotypic measures when comparing the various MDFs to the control group in the low dose mannan trial.

1.2 Feed-associated microbiota

To establish a baseline for our analysis, we used 16S rRNA amplicon sequencing to profile the microbial composition of the different feeds before consumption. The mannan supplemented feeds and the control feed used in the low dose trial contained 13 phyla. Firmicutes (CTR: 52.68%, MC1: 51.8%, MC2: 49.94%, MN3: 48.73%) and Proteobacteria (CTR: 43.45%, MC1: 44.22%, MC2: 46.19%, MN3: 46.63%) emerged as the dominant phyla, followed by Fusobacteria (CTR: 3.31%, MC1: 2.97%, MC2: 3.43%, MN3: 4.06%). The remaining phyla combined represented 1% of the relative abundance (**Supplementary Figure 2A**). At the genus level, the predominant genus was *Photobacterium* (CTR: 37.9%, MC1: 38.9%, MC2: 40.53%, MN3: 4.06%) and lactobacillus (CTR: 38.93%, MC1: 38.54%, MC2: 39.39%, MN3: 39.45%). Additional genera belonging to *Moritella*, *Tepidimicrobium*, and *Tepidimicrobium* were also identified, although they exhibited lower abundance (**Supplementary Figure 2B**). Alpha and beta diversity showed no significant changes between the mannan-supplemented diets and the control feed (**Supplementary Figure 2C-E**).

For the feed used in the high dose β-mannan trial, Proteobacteria (54.44%) were less abundant in the experimental feed than in the control feed (63.37%) while Firmicutes (38.61%) were more abundant in the experimental feed than in the control feed (31.03%) (**Supplementary Figure 3A**). Fusobacteriota and Bacteroidota were also more abundant in the β-mannan diet (3.46% and 2.07%) than the control (2.81% and 1.12%). The remaining phyla combined represented 1% of the relative abundance. Notably, the β-mannan feed exhibited higher relative abundances of *Lactobacillus*, *Limosilactobacillus,* and *Moritella* compared to the control (**Supplementary Figure 3B**). Our analysis of alpha and beta diversity revealed differences between control and experimental feed. Alpha-diversity indicated higher bacterial diversity in the β-mannan feeds (median richness 159) compared to the control (median richness 128) using Observed richness and Shannon diversity index (**Supplementary Figure 3C-D**). Beta-diversity highlighted distinct clustering patterns between the feed and control samples, indicating the presence of different microbial communities (**Supplementary Figure 3E**).

1.3 Low mannan inclusion rates have little effect on gut microbial community structure and function

A total of 839 bacterial genera from 44 phyla were detected by 16S rRNA sequencing in the gut content of fish fed mannan feeds at low dose. The most abundant phyla were Proteobacteria (54.43%), Firmicutes (32.06%), Bacteroidota (3.84%), Actinobacteriota (2.15%), and Chloroflexi (1.23%). The remaining 39 phyla combined represented 1% of the relative abundance (**Supplementary Figure 4A**). Taxonomic identification at the class, family, and genus levels revealed that most of the Proteobacteria belonged to the class Gammaproteobacteria (33%), with varying amounts of the families Vibrionaceae (7.80%), Burkholderiaceae (6.81%) and

Pseudomonadaceae (5.23%). Among these, the most abundant genera were Lactobacillus (18.16%), Photobacterium (7.63%), Burkholderia_Caballeronia_Paraburkholderia (BCP group) (5.94%), and Pseudomonas (5.22%). We also observed a high abundance of unclassified bacterial genera belonging to order Rickettsiales (16.88%) (**Supplementary Fig. 4B-D**).

We explored the effect of developmental stages on the microbiome diversity and found a significant decrease in bacterial diversity as time progressed. Specifically, we found that the bacterial diversity was highest in early developmental stages (pre-smolts: T1) and decreased over time (**Supplementary Fig. 5**). Our findings revealed significant differences in gut microbiota between sampling time points (PERMANOVA for Bray-Curtis, p = 1e-04, r2 = 0.0386) (**Fig. 1D**), but not between diets (**Fig. 1E**). Gut content samples from pre-smolts (T1) were collected before seawater transfer and displayed similar bacterial diversity compared to samples from post-smolts (T3). We obtained similar results using weighted and unweighted UniFrac matrices (data not shown).

In addition to exploring overall bacterial diversity, we performed statistical analyses to identify specific bacterial genera that exhibited significant variations across different diets and developmental stages compared to the control group. Only in samples from smolts (T2), we observed a significant change in the abundance of certain bacterial genera in response to dietary changes (**Fig. 1G**). Bacterial genera belonging to the *BCP* group, *Herbaspirillum, Nitrospirillum,* and *Cupriavidus* showed significant increases in abundance when salmon were fed the MC2 and MN3 diet, as compared to the control group (Wilcoxon test, adjusted $p<0.05$) (**Fig. 1G**).

Metatranscriptomic analysis after mapping reads to SMGA database showed 205,322 genes expressed in the salmon hindgut across the four life stages (**Supplementary Figure 6**). Changes in the number of expressed bacterial genes were detected

between stages, where T1 showed a higher number of expressed genes (n=33,548) than T2 and T3 (n=14,656 and n=23,185, respectively) (**Supplementary Figure 6**). Members of the genera *Lactobacillus, Limosilactobacillus, Sphingomonas, Pseudomonas, Cetobacterium, Glutamicibacter, Serratia, Photobacterium*, and *Paraburkholderia* were among the most metabolically active bacteria. Among them, *Serratia liquefaciens* S38_bc38 showed expression of a GH18 chitinase for potential hydrolysis of chitin, as well genes encoding CAZymes (GH3 β-glucosidase and GH31 α-xylosidase) for utilization of xyloglucan-derived oligosaccharides. *Photobacterium* sp. A22_bc66 expressed genes putatively involved in the complete depolymerization of chitin (GH18 chitinase and GH20 beta-hexosaminidase) and cellulose (GH3 β-glucosidase and GH9 cellulase). *Paraburkholderia fungorum* expressed genes for potentialdepolymerization of cello-oligosaccharides (GH3), manno-oligosaccharides (GH2 β-mannosidase/β-glucosidase/β-galactosidase) and linear pectin galactan-derived oligosaccharides (GH42 β-galactosidase). Lactic acid bacteria *Lactobacillus kitasatonis* and *Limosilactobacillus mucosae* expressed genes CAZymes involved in the putative depolymerization of pectic galactans (GH36 α-galactosidase, GH42 β-galactosidase, GH43 arabinofuranosidase) and xyloglucans (GH2 β-galactosidase, GH3 β-glucosidase, GH31 α-xylosidase, GH43 arabinofuranosidase), eventually resulting in production of lactate (**Fig. 3, Supplementary Data 4d**). *Sphingomonas* showed expression of a GH9 cellulase, GH30 xylanase, GH33 sialidase, and GH125 exo-alpha-1,6-mannosidase, which could play a role in the depolymerization of xylans and salmon mucin. *Pseudomonas_E yamanorum* expressed genes for potential hydrolysis of salmon mucin (GH33 sialidase, GH109 alpha-N-acetylgalactosaminidase) as well as for depolymerization of beta-glucans' backbone (GH74) and further processing of the resulting cellobiose into glucose via a GH3

representative. Additionally, *Glutamicibacter* sp. S15_bc15 expressed genes encoding GH3 representatives for putative degradation of cello-oligosaccharides, while *Cetobacterium* expressed genes coding for a lacto-N-biose phosphorylase GH112 (**Fig. 3, Supplementary Data 4d**).

Furthermore, differential expression analysis for individual microbial genes (DEGs) using *de novo* metatranscriptomics showed limited significant changes in gene expression between the β-mannan-supplemented diets and the control group at each life stages; the number of DEGs ranged from 8 to 36 out of 117,261 microbial genes in the three different experimental groups. The differentially expressed genes coded for enzymes putatively involved in processes related to bacterial cell growth. No expressed gene encoding GH26 endo β-1,4-mannanases for cleavage of the polymeric β-mannan (in MN3) or GH76 α-1,6-mannanases for hydrolysis of α-mannans (in MC1 and MC2) could be detected in the metatranscriptomes (**Supplementary Data 4e**).

1.4 Low mannan inclusion rates have little effect on salmon gene regulation and physiology

To understand host responses to the mannan-supplemented diets, we performed transcriptomics analysis on the gut epithelium. Significant differences in gene expression were found between life stages (PERMANOVA for Euclidean, $p = 1e\text{-}04$, $r2 = 0.367$), with different sampling time forming distinct clusters in principle component analysis (**Supplementary Figure 7**). However, we did not observe any corresponding clustering of the experimental diets (**Fig. 1F**). We further performed differential expression analysis for individual genes (DEGs) and observed none or few significant changes in gene expression between the mannan-supplemented diets and the control group at each time point. Further analysis shows that a few samples were

disproportionately influencing these DEGs. Following the exclusion of these samples (considered outliers), no statistically significant changes were observed. Therefore, we cannot confidently consider these genes as reliable indicators.

1.5 A high inclusion β-mannan diet affects the composition of the gut microbiota but has limited effects on salmon metabolism

In a second trial performed with a acetylated galactoglucomannan inclusion level of 4% (referred to as 4%MN3), we broadened our sampling scope to include both the hindgut (lower GIT) and pyloric caeca; indeed, analysis of pyloric caeca contents could provide additional insights into whether the β-mannan is metabolized and induce microbial compositional changes in the upper part of the salmon GIT.

We employed a phenotypic scoring analysis to assess the effects of 4%MN3 diet on parameters including weight, length, gutted weight, condition factor, hepatosomatic index, cardio somatic index, and organ integrity for the fish (**Supplementary Data 4f**). There were no significant differences in any of the phenotypic measures when comparing data from the experimental to the control group.

Amplicon sequencing analysis of hindgut samples revealed the presence of 683 bacterial genera from 36 different phyla, whereas the pyloric caeca samples contained 510 bacterial genera from 33 phyla. Among these samples, the most abundant phyla observed in the hindgut were Firmicutes (85.94%), Proteobacteria (9.78%), and Actinobacteriota (2.26%). In the pyloric caeca, the dominant phyla were Firmicutes (64.83%), Proteobacteria (29.10%), and Actinobacteriota (2.16%). The remaining 34 phyla in the hindgut and 30 phyla in the pyloric caeca combined represented 1% of the relative abundance. Notably, the most prevalent genera in the hindgut were *Limosilactobacillus* (15.69%), *Peptostreptococcus* (15.61%), and *Lactobacillus* (14.47%), whereas the *BCP* group (15.57%) dominated the pyloric caeca, followed by

Limosilactobacillus (11.04%), *Peptostreptococcus* (10.81%), and *Lactobacillus* (10.78%) (**Supplementary Figure 8**).

Our analysis revealed a significant increase in the Shannon (Wilcoxon test, p=0.045) diversity in the hindgut samples from the fish given the 4%MN3 diet compared to the control group (**Fig. 2C**). However, no statistically significant alterations were observed in the alpha diversity in the pyloric caeca samples. We explored the dissimilarity between microbial communities using Bray-Curtis distance metrics tested by PERMANOVA for diet. The results show shifts in the microbial composition for both hindgut and pyloric caeca samples when comparing the β-mannan diet group to the control group. These shifts were statistically significant (hindgut: p = 0.013, R2 = 0.093 and pyloric caeca: p = 0.0042, R2 = 0.087) (**Fig. 2D, E**), suggesting that the 4%MN3 diet not only influenced the diversity of the microbial species within these samples but also led to substantial changes in their overall composition. Differential abundance analysis at the genus showed that genera belonging to the *BCP* group (Wilcoxon, p<0.001) and *Pseudomonas* (Wilcoxon, hindgut: p<0.001, pyloric caeca: p<0.05) were significantly more abundant in both hindgut and pyloric caeca samples from fish fed the 4%MN3 diet, whereas *Limosilactobacillus* was less abundant (Wilcoxon, p<0.001) (**Fig. 2F**).

1.6 A high inclusion β-mannan diet has limited effects on salmon metabolism

We observed 47,563 genes with no clear clustering of the sample groups in the host gene expression data from the hindgut tissue (**Fig. 2G**). We did however observe significant increase in the expression of three genes: dynein heavy chain-like, SH3 domain binding glutamate-rich protein like 2 and the third one is an uncharacterized gene (**Supplementary Data 4g**). We also identified significant downregulated

expression in three genes: contactin-associated protein-like 2, prosaposin receptor GPR37, and heterogeneous nuclear ribonucleoprotein C-like.

Similar to hindgut, we observed 47,433 genes with no clear clustering of the sample groups in the pyloric caeca tissue (**Supplementary Figure 9**). Furthermore, we observed downregulated expression in two genes: NLR family CARD domain-containing protein 3-like and an uncharacterized gene (**Supplementary Data 4g**). Despite a set of discernible host gene expression changes associated with the 4%MN3 diet compared to control fed fish in the hindgut and pyloric caeca, none of these genes can be definitively linked to beta-mannan degradation.

1.7 A high inclusion β-mannan diet has limited impact on the functions of the salmon gut microbiome

Metatranscriptomic analysis after mapping reads to SMGA database of the hindgut content identified 62,065 bacterial genes expressed in both control and the 4%MN3 group (**Supplementary Figure 10**). While there were no significant changes in the gene expression levels (TPM) between the control and experimental group, members of the orders Pseudomonadales, Enterobacterales, Burkholderiales, Rhizobiales, Sphingomonadales, and Lactobacillales were among the most active populations (**Supplementary Figure 10**). The top five genera with the highest number of expressed genes were *Pseudomonas*, *Paraburkholderia*, *Aeromonas*, *Sphingomonas*, and *Limosilactobacillus*. Given that the amplicon sequencing analysis revealed that the BCP group, and in particular *Paraburkholderia*, was present at higher abundance in the hindgut of fish fed high-dose β-mannan, we explored the differential expression of CAZyme-encoding genes. However, no gene coding for CAZymes potentially involved in mannan degradation (GH2 β-mannosidase, GH5 and GH26 β-

mannanases, or GH36 α-galactosidase) were detected in the *Paraburkholderia* transcriptome (**Supplementary Data 4h**).

Differential expression analysis for individual microbial genes (DEGs) using genes recovered by *de novo* metatranscriptomic assembly showed limited significant changes in gene expression between the 4%MN3 and the control group. Only 5 statistically significant DEGs from 17,094 microbial genes were detected, none of which were metabolically linked with β- mannan (**Supplementary Data 4i**).

1.8 A high inclusion β-mannan diet has limited effects on gut SCFAs

Hindgut and pyloric caeca contents were also analyzed for the presence of 10 SCFAs that is acetic acid, formic acid, propanoic acid, 2-methyl-propanoic acid, 3-methylbutanoic acid, pentanoic acid, 4-methyl-pentanoic acid, hexanoic acid, and heptanoic acid (**Supplementary Data 4j**). Among these compounds, only acetic acid was detected above the threshold for more than half of the samples and exhibited a significant increase in both hindgut and pyloric caeca of fish fed the 4%MN3 diet when compared to the control samples. However, it must be noted the MN3 substrate used in the high dose β-mannan trial is acetylated; therefore, we cannot rule out that increased acetate levels are in fact attributed to deacetylation of the β-mannan due to the acidic pH of the gut environment and thus might not be the result of microbial fermentation.

Supplementary figures

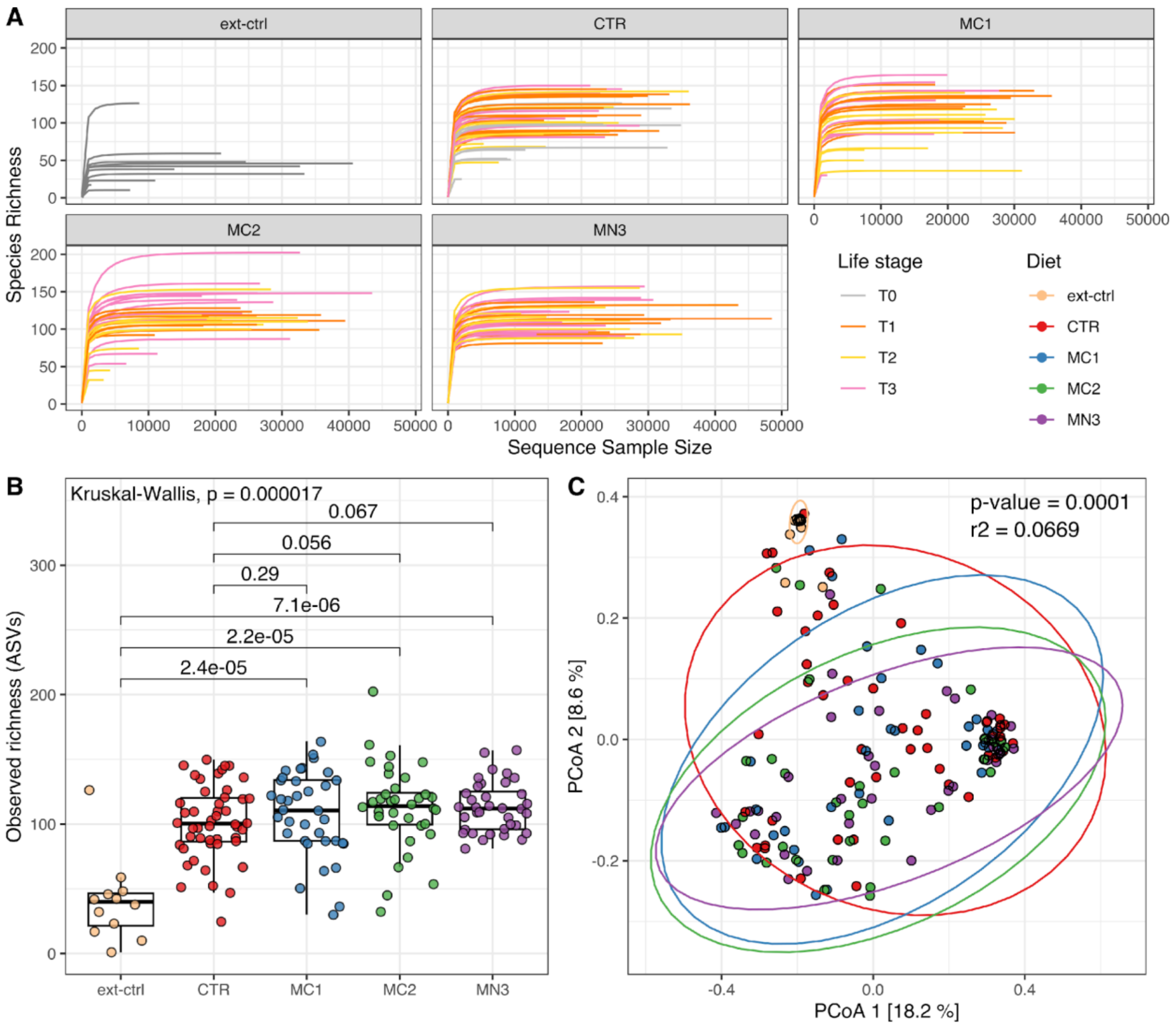

Supplementary Figure 1. Microbiota composition at ASV level detected in the feeds administered to salmon in the low dose mannan trial. Atlantic salmon was fed either a control (CTR) diet or three experimental diets (MC1, MC2, and MN3) including different versions of β-mannans at 0.2% inclusion levels. Extraction controls (ext-ctrl) samples were included in the analysis. (**A**) Rarefaction curves calculated using the richness index at increasing sequencing depth for each feed type and for the extraction control samples. Curves are colored according to the sample collection time point, as indicated in the legend. T0 indicates the trial starting point, followed by T1 (pre-smolts), T2 (smolts) and T3 (post-smolts). (**B**) Alpha-diversity for observed richness tested using the Kruskal-Wallis test. The Benjamini-Hochberg FDR method was used for p-value correction (p-value <0.05). Wilcoxon test was

performed to determine which group of the independent variable differs from each other group. (**C**) Principal coordinates analysis (PCoA) plot showing beta-diversity analysis by sample (diet) prior to removal of contaminant ASVs using the decontam package (see methods section). Beta-diversity was assessed based on Bray-Curtis dissimilarity and tested by PERMANOVA.

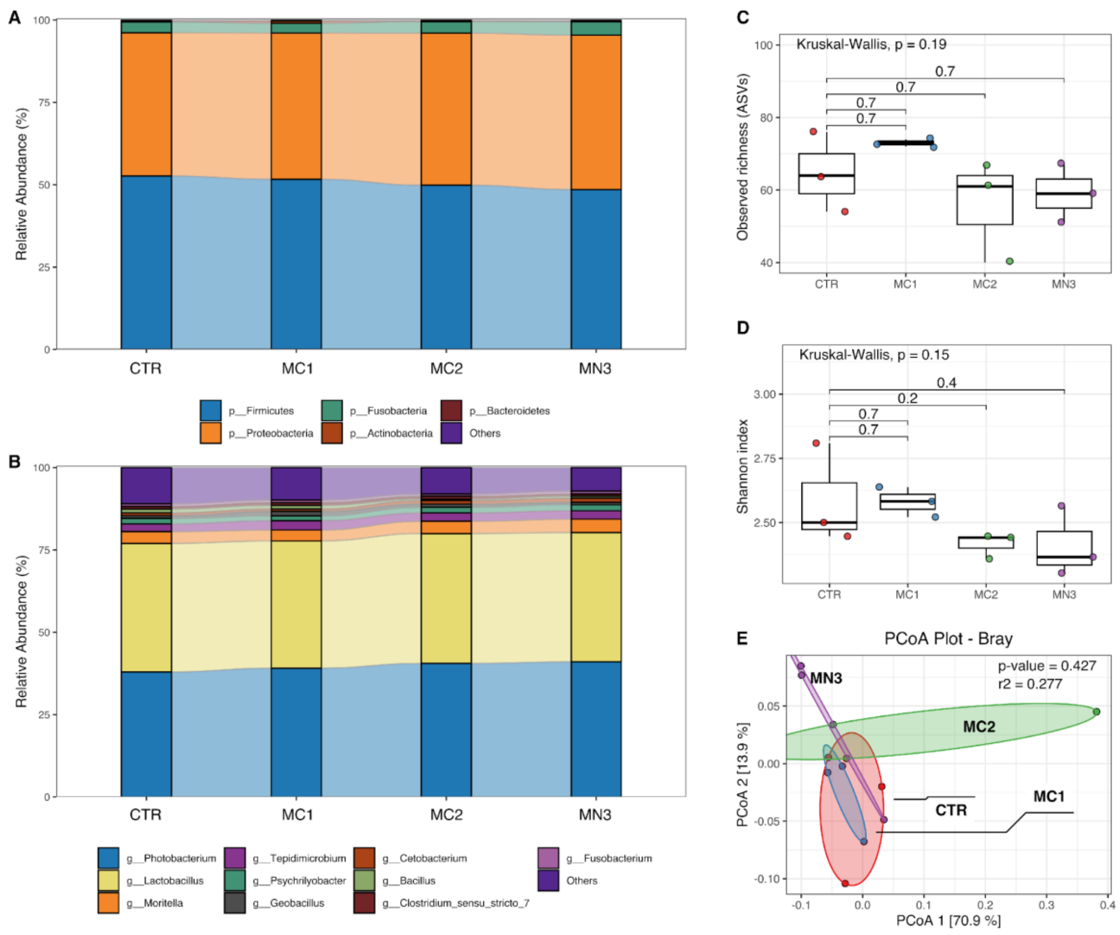

Supplementary Figure 2. Composition of feed-associated microbiota detected in the feeds administered in the low dose mannan trial. Atlantic salmon received either a control (CTR) diet or three experimental diets (MC1, MC2, and MN3) supplemented with different versions of β-mannans (**A**) Top 5 most abundant phyla detected in hindgut contents (average total abundance > 1% or at least in one sample). Other bacterial phyla are included as "Others". Samples are grouped by feed group and each bar plot shows the average relative abundance of phyla per feed group. (**B**) Top 10 most abundant genera (average total abundance > 1% or at least in one sample). Other bacterial genera are grouped into "Others". Samples are grouped by feed group and each bar plot shows the average relative abundance of genera per feed group. (**C**) Alpha-diversity for feed samples calculated using observed richness index. Alpha-diversity was tested using the Kruskal-Wallis test, and Benjamini-Hochberg FDR method was used for p-value correction (p-value <0.05). For pairwise comparison, a Wilcoxon test was performed. (**D**) Alpha-diversity for feed samples calculated using the Shannon diversity index. Alpha-diversity was tested using the Kruskal-Wallis test, and Benjamini-Hochberg FDR method was used for

p-value correction (p-value <0.05). For pairwise comparison, a Wilcoxon test was performed. (**E**) Principal coordinates analysis (PCoA) plot showing beta-diversity calculated by Bray–Curtis distance.

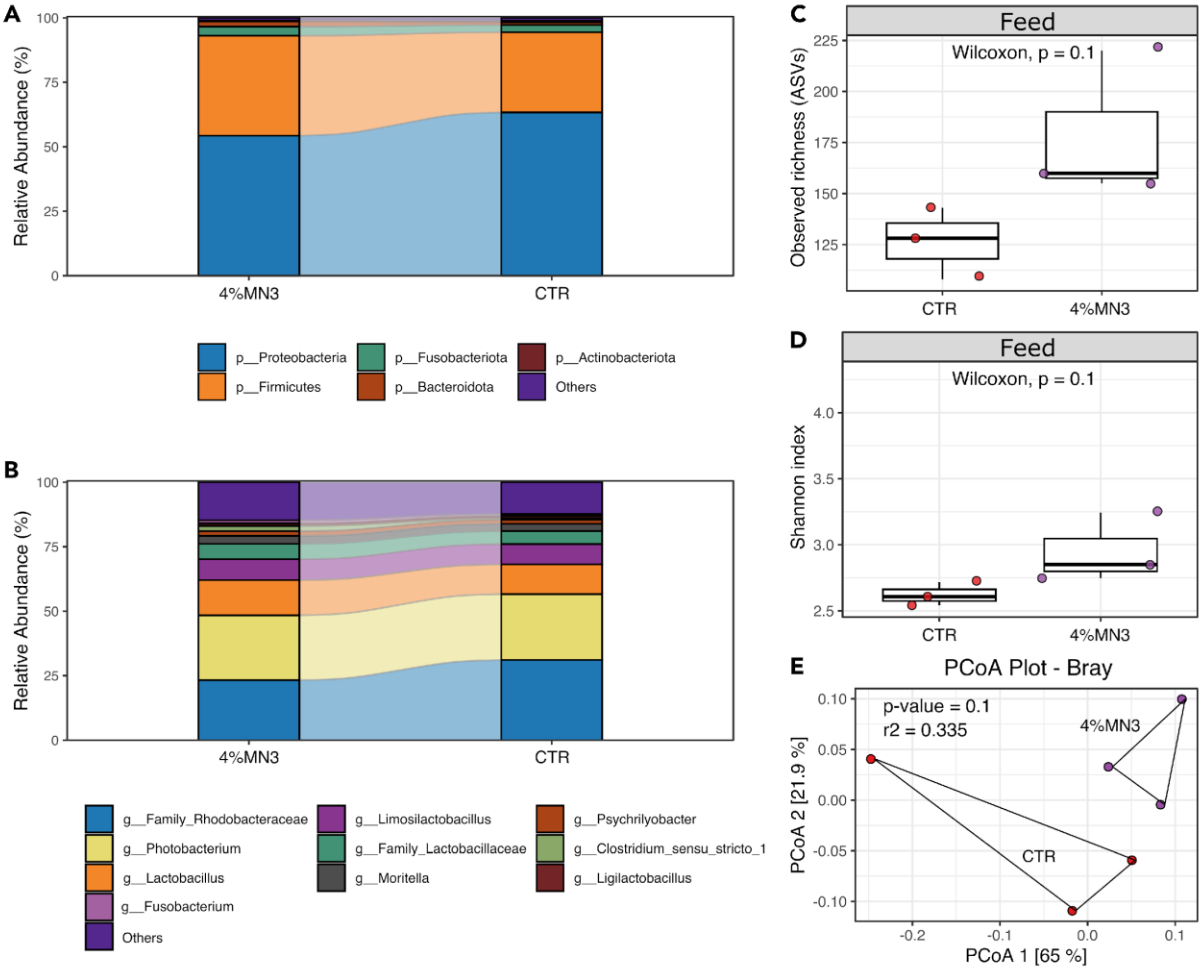

Supplementary Figure 3. **Microbiota composition detected in the feeds used in the high dose β-mannan trial.** Atlantic salmon was fed either a control (CTR) diet or an experimental diet supplemented with 4% β-mannan (4%MN3) (**A**) Top 5 most abundant phyla (average total abundance > 1% or at least in one sample). Other bacterial phyla are grouped into "Others". Samples are grouped by feed group and each bar plot shows the average relative abundance of phyla per feed group. (**B**) Top 10 most abundant genera (average total abundance > 1% or at least in one sample). Other bacterial genera are grouped into "Others". Samples are grouped by feed group and each bar plot shows the average relative abundance of genera per feed group. (**C**) Alpha-diversity for feed samples calculated using the richness index. For pairwise comparison, a Wilcoxon test was performed. (**D**) Alpha-diversity for feed samples calculated using Shannon diversity index. For pairwise comparison, a Wilcoxon test was performed. (**E**) Principal coordinates analysis (PCoA) plot showing beta-diversity calculated using Bray-Curtis distance.

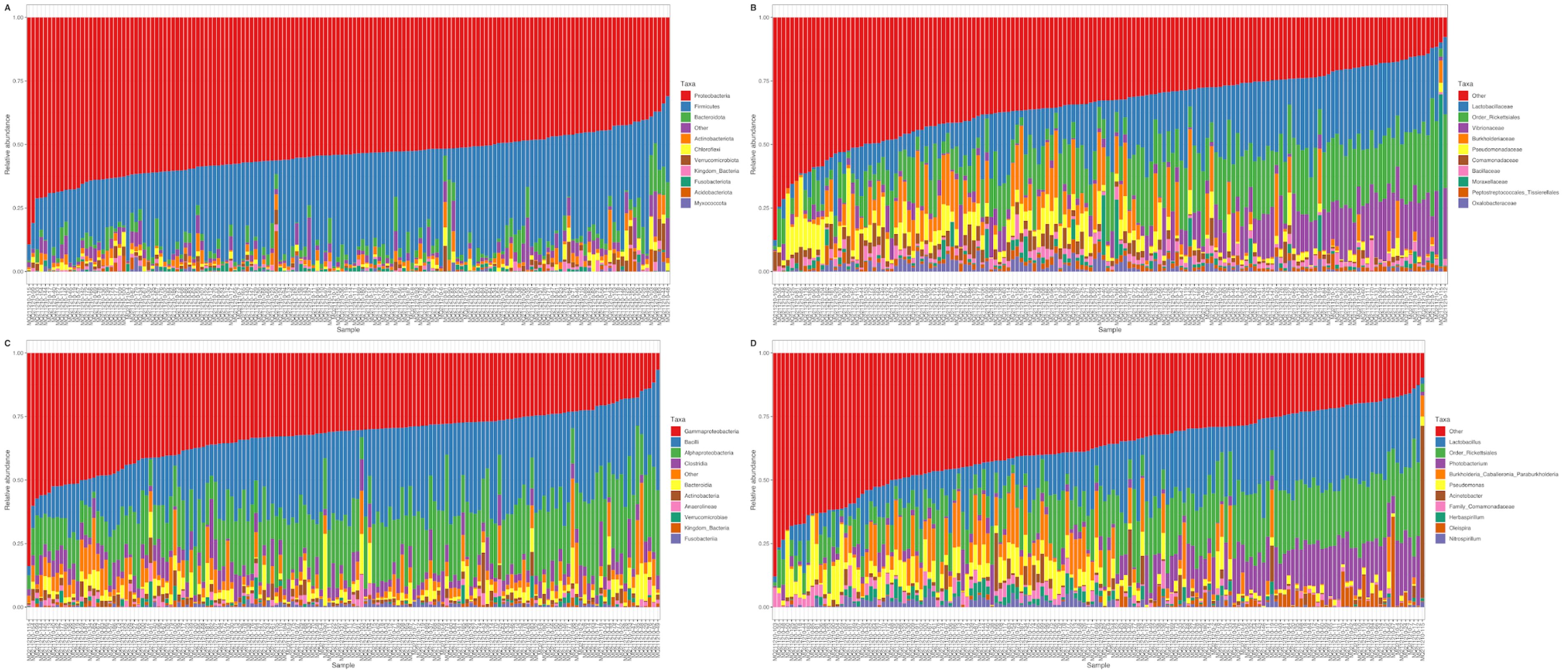

Supplementary Figure 4. Bacterial microbiome by 16S rRNA gene (panels A–D) at four taxonomic levels (a) phylum (b) class (c) family, and (d) genus in hindgut samples from fish fed a diet supplemented with varying β-mannans. Each of the above-stacked bar plots illustrates the average relative abundance (y-axis) of the microbiota at different taxonomic levels. Taxa with a mean abundance of at least 1% across all samples are represented in colors; those with < 1% abundance are not shown. Each column represents one individual. The taxonomy for bacterial ASVs was assigned from the SILVA database.

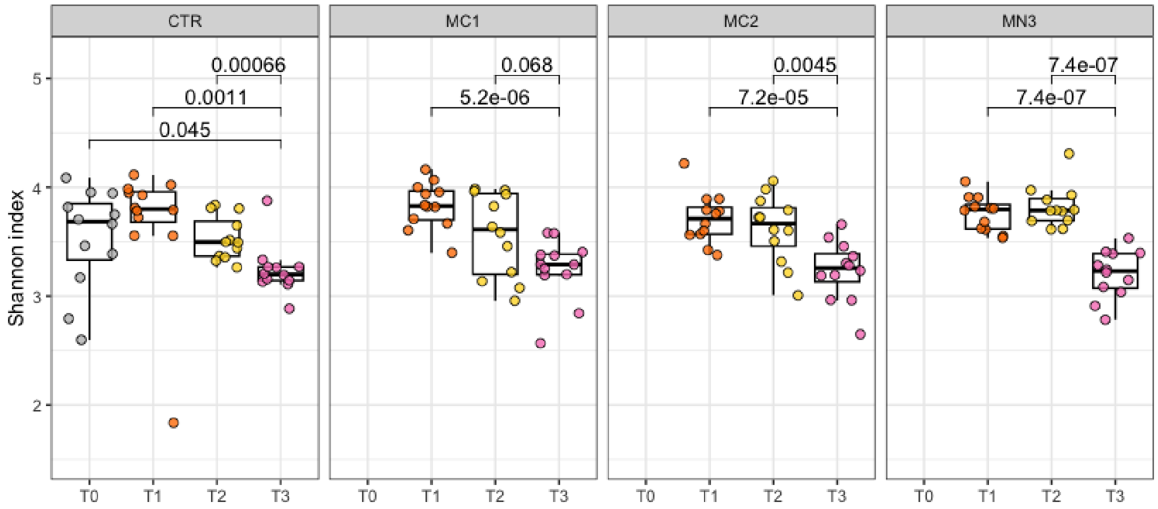

Supplementary Figure 5: Hindgut microbiome composition of fish fed diets supplemented with varying β-mannans across different developmental stages. Alpha-diversity was computed using the Shannon index and statistically tested using the Wilcoxon test. P-values were corrected using the Benjamin-Hochberg FDR method (p-value <0.05). Samples are grouped by feed group with CTR, MC1, MC2, and MN3 representing distinct diets (CTR: control group; MC1, MC2, and MN3 denote three diets supplemented with different versions of β-mannans at 0.2% inclusion level). In each subpanel, T0 indicates the trial starting point (parr), followed by T1 (pre-smolts), T2 (smolts) and T3 (post-smolts).

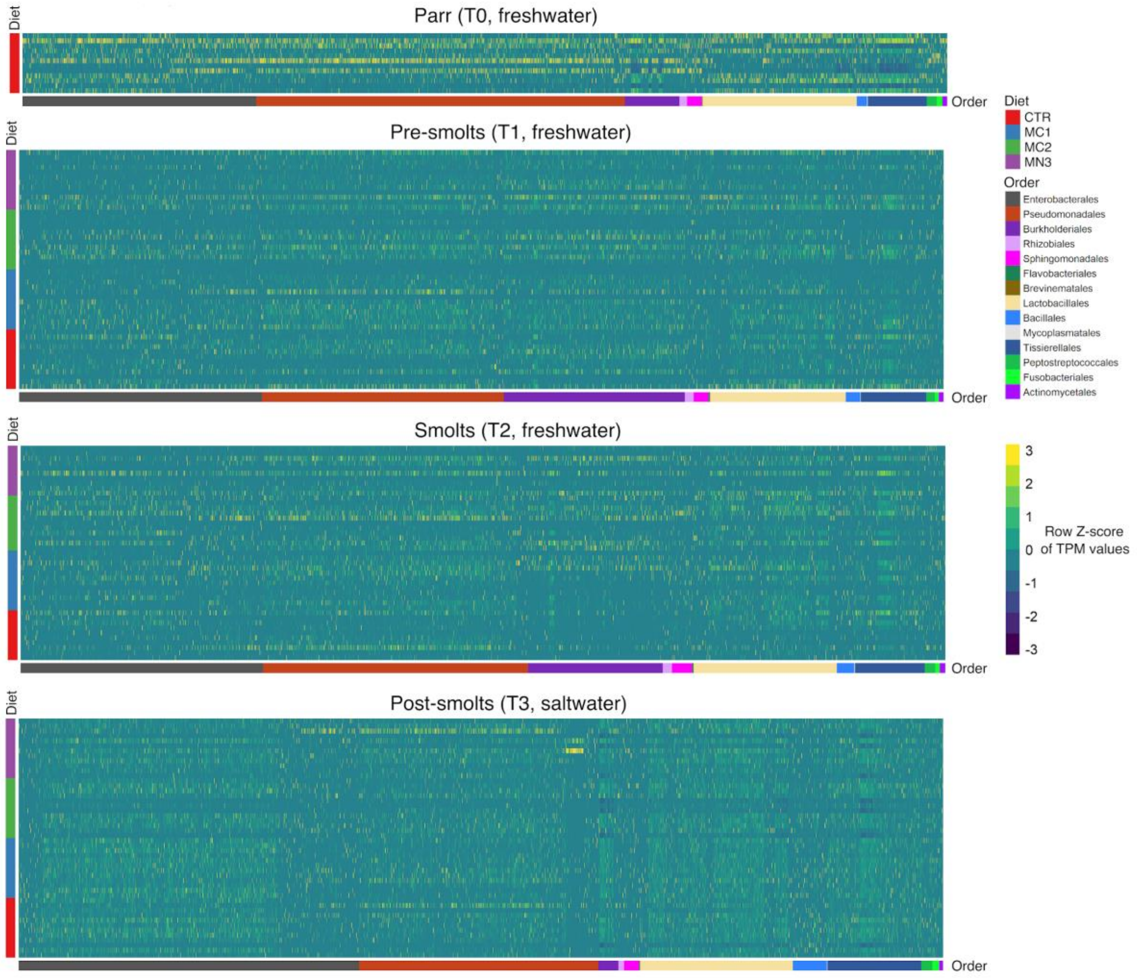

Supplementary Figure 6. Heatmap illustrating the metatranscriptomes from hindgut content of salmon fed either a control diet or experimental diets supplemented with β-mannans. Variation in gene expression of all bacterial genes (x-axis) in metatranscriptomes generated from hindgut samples obtained from fish and collected at different life stages (y-axis). T0 (parr, freshwater), T1 (pre-smolts, freshwater), T2 (smolts, freshwater), T3 (post-smolts, seawater).

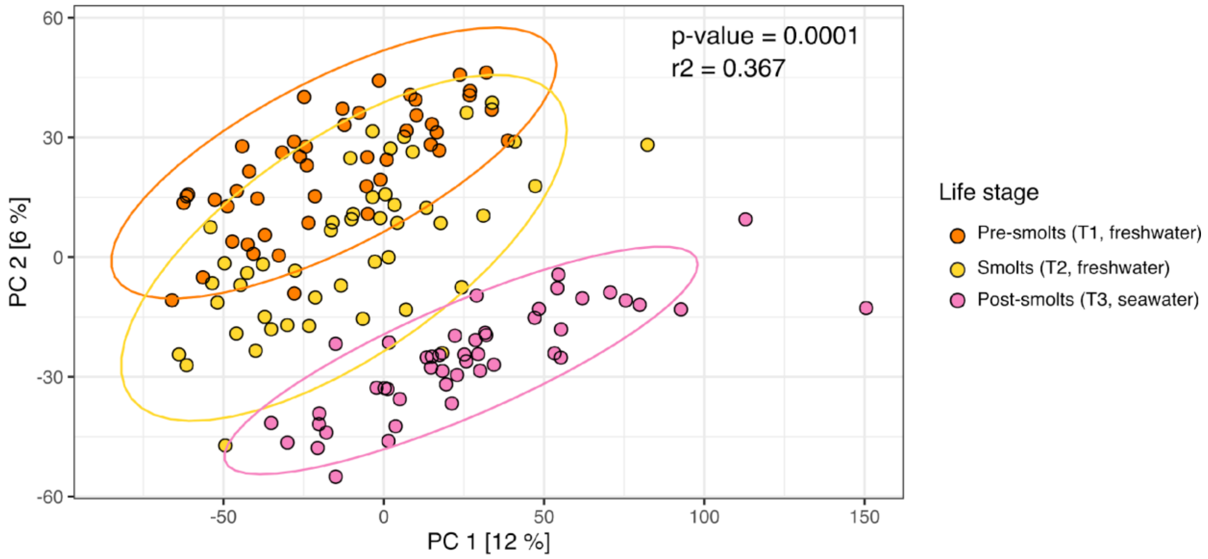

Supplementary Figure 7. Principal Component Analysis (PCA) plot of host genes expressed in all samples and feeding groups at different salmon developmental stages. Each dot on the plot represents an individual sample, and the color indicates the fish life stage. P values were calculated using adonis and 9999 permutations.

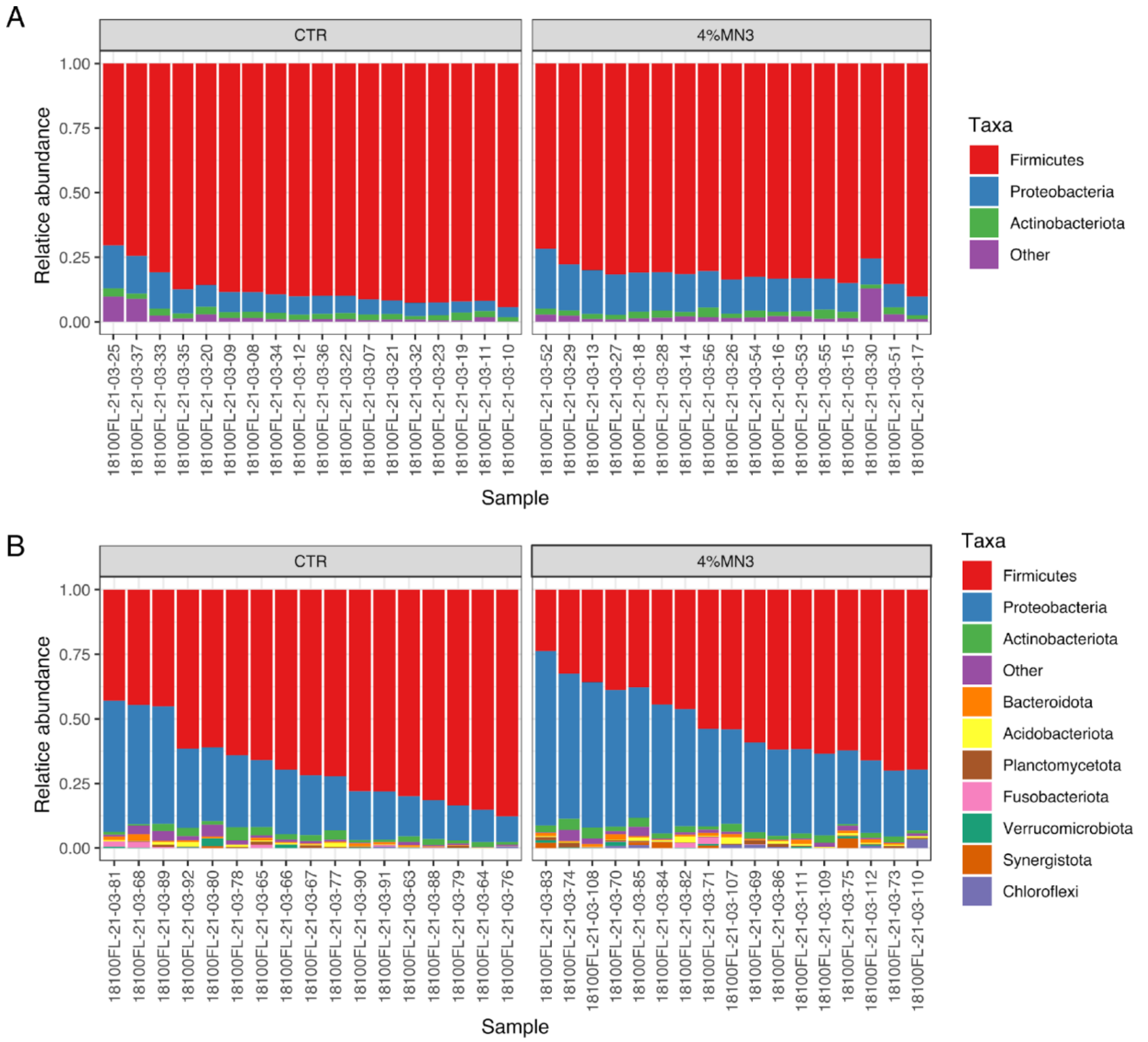

Supplementary Figure 8. Most abundant phyla in pyloric caeca and hindgut samples from fish fed a diet supplemented with a high dose of β-mannan . Top 3 most abundant phyla and top 10 most abundant genera detected in digesta from (**A**) hindgut and (**B**) pyloric caeca from fish fed either a control (CTR) or the experimental diet supplemented with 4% β-mannan (4%MN3). Taxa with a mean abundance of at least 1% across all samples are represented; taxa with < 1% abundance are grouped as “Other”. Each column represents one individual salmon.

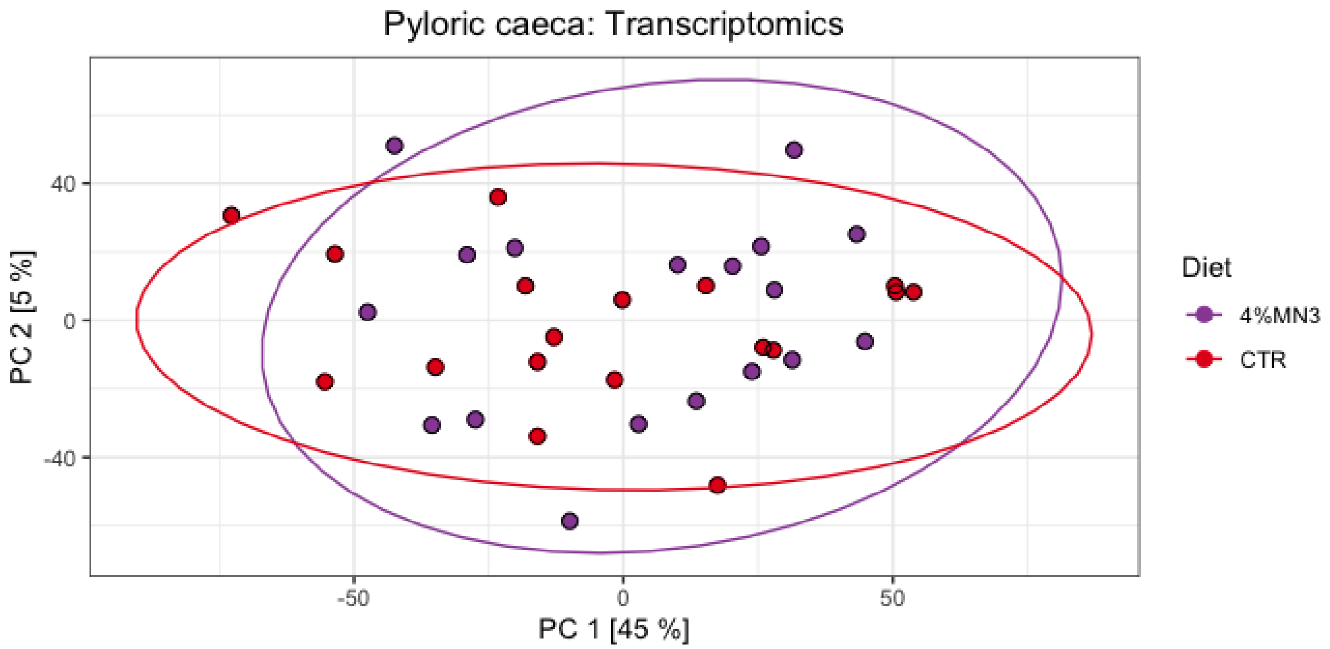

Supplementary Figure 9. Principal Component Analysis (PCA) plot of host genes expressed in all samples for pyloric caeca. Each dot on the plot represents an individual sample, and the color indicates the fish fed either control (CTR) or 4% β-mannan (4%MN3).

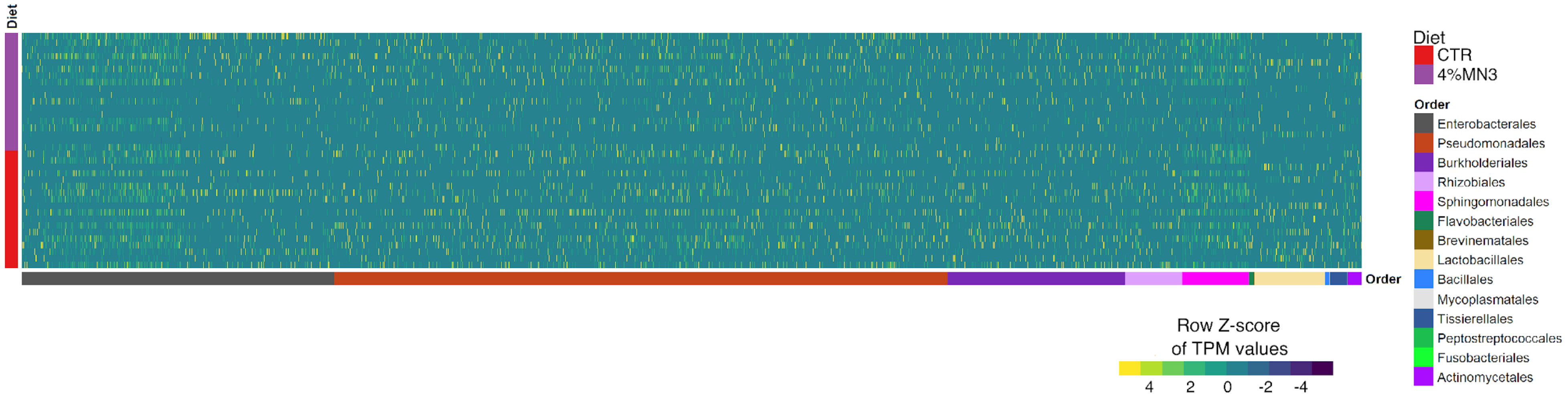

Supplementary Figure 10. Heatmap illustrating the metatranscriptomes from hindgut content of salmon fed either the control or the 4%MN3 diet. Variation in gene expression of all bacterial genes (x-axis) in metatranscriptomes generated from hindgut samples obtained from fish fed different diet (y-axis).
