# Supplementary material for: The need for high-resolution gut microbiome characterization to design efficient strategies for sustainable aquaculture production
Source: Commun Biol. 2024 Oct 25;7:1391. doi: 10.1038/s42003-024-07087-4 (PMC11511968; doi:10.1038/s42003-024-07087-4)
Supplement: Supplementary file 2 — Description of Additional Supplementary Files [file 42003_2024_7087_MOESM2_ESM.pdf]

Description of Additional Supplementary Files

File name: Supplementary Data 1

Description: The source data behind Figure 1.

File name: Supplementary Data 2

Description: The source data behind Figure 2.

File name: Supplementary Data 3

Description: Expression levels of salmon genes detected in transcriptomic data.

File name: Supplementary Data 4

Description:

Data 4a- Formulation and calculated chemical composition of the experimental feeds used in the low dose mannan trial.

Data 4b- Formulation and chemical composition of the experimental feeds used in the high dose mannan trial

Data 4c- Growth and biometric traits of Atlantic salmon fed control diet or low dose mannan diets.

Data 4d- Annotated genes for salmon gut microbiome metabolic features.

Data 4e- Differential microbial gene expression analysis in hindgut samples of salmon fed control and experimental diets supplemented with β-mannans.

Data 4f-Growth and biometric traits of Atlantic salmon fed control diet or a high dose β-mannan diet (4%MN3).

Data 4g- Gene expression patterns in hindgut and pyloric caeca samples for fish fed a diet supplemented with a high-dose β-mannan (4%MN3).

Data 4h- Carbohydrate-Active Enzyme (CAZy) Genes from hindgut content of salmon fed either the control or the 4%MN3 diet.

Data 4i- Differential microbial gene expression analysis in hindgut samples of salmon fed control and 4% β-mannan (4%MN3).

Data 4j- Short-Chain Fatty Acids detected in hindgut and pyloric caeca content from fish receiving either a control or a diet supplemented with 4% β-mannan (4%MN3).
