# Supplementary material for: The need for high-resolution gut microbiome characterization to design efficient strategies for sustainable aquaculture production
Source: Commun Biol. 2024 Oct 25;7:1391. doi: 10.1038/s42003-024-07087-4 (PMC11511968; doi:10.1038/s42003-024-07087-4)
Supplement: Supplementary file 7 — Reporting Summary [file 42003_2024_7087_MOESM7_ESM.pdf]

nature portfolio

Corresponding author(s): Sabina Leanti La Rosa
Torgeir R. Hvidsten

Last updated by author(s): Oct 5, 2024

Reporting Summary

Nature Portfolio wishes to improve the reproducibility of the work that we publish. This form provides structure for consistency and transparency in reporting. For further information on Nature Portfolio policies, see our Editorial Policies and the Editorial Policy Checklist.

Statistics

For all statistical analyses, confirm that the following items are present in the figure legend, table legend, main text, or Methods section.

n/a	Confirmed	
☐	☒	The exact sample size (*n*) for each experimental group/condition, given as a discrete number and unit of measurement
☐	☒	A statement on whether measurements were taken from distinct samples or whether the same sample was measured repeatedly
☐	☒	The statistical test(s) used AND whether they are one- or two-sided<br>*Only common tests should be described solely by name; describe more complex techniques in the Methods section.*
☒	☐	A description of all covariates tested
☒	☐	A description of any assumptions or corrections, such as tests of normality and adjustment for multiple comparisons
☐	☒	A full description of the statistical parameters including central tendency (e.g. means) or other basic estimates (e.g. regression coefficient) AND variation (e.g. standard deviation) or associated estimates of uncertainty (e.g. confidence intervals)
☐	☒	For null hypothesis testing, the test statistic (e.g. *F*, *t*, *r*) with confidence intervals, effect sizes, degrees of freedom and *P* value noted<br>*Give P values as exact values whenever suitable.*
☒	☐	For Bayesian analysis, information on the choice of priors and Markov chain Monte Carlo settings
☒	☐	For hierarchical and complex designs, identification of the appropriate level for tests and full reporting of outcomes
☒	☐	Estimates of effect sizes (e.g. Cohen's *d*, Pearson's *r*), indicating how they were calculated

Our web collection on statistics for biologists contains articles on many of the points above.

Software and code

Policy information about availability of computer code

Data collection

No software was used for data collection.

Data analysis

All software packages used are described in the Methods section and are publicly or commercially available. They include:
- for 16S rRNA amplicon sequencing analysis: QIIME2 (qiime2-2021.8) pipeline, Silva database (release 138) for taxonomy assignment, phyloseq package (v1.42.0), ggplot2 (v3.4.1), vegan package (v2.6.4), R (v4.2.2 and v4.2.3).
- for host transcriptomics analysis: FastQC (v0.11.3), AdapterRemoval (v2.1.3), STAR (v2.7.2), DESeq2 Bioconductor R package (v1.38.3).
- for metatranscriptomics analysis: fastp (v0.12.4), SortMeRNA (v4.3.482), STAR (v2.7.2), kallisto (v0.44.0), Bioconductor tximport 1.26.1, Megahit (v1.2.9), DRAM (v1.3), Bioconductor tximport (v1.26.1), DESeq2 Bioconductor R package (v1.38.3)
- for metabolomics: ChromaTOF® (LECO), Chemstation (Agilent), PARADISe software, MetaboAnalyst version 5.0

All mentioned tools used for the data analysis in this study are publicly available, and the version and parameters used have been indicated. Custom scripts are available at https://github.com/shashank-KU/ImprovaFish-MDF-Effects

For manuscripts utilizing custom algorithms or software that are central to the research but not yet described in published literature, software must be made available to editors and reviewers. We strongly encourage code deposition in a community repository (e.g. GitHub). See the Nature Portfolio guidelines for submitting code & software for further information.

Data

Policy information about availability of data

All manuscripts must include a data availability statement. This statement should provide the following information, where applicable:

- Accession codes, unique identifiers, or web links for publicly available datasets
- A description of any restrictions on data availability
- For clinical datasets or third party data, please ensure that the statement adheres to our policy

The raw metagenomics dataset analyzed during the current study for low and high dose trials have been deposited in the Sequence Read Archive (SRA) repository under project id PRJNA947090. The raw host transcriptomics and metatranscriptomics data for the low dose mannan trial are available under project id PRJEB73366 and PRJEB67787, and for the high dose mannan trial under project id PRJNA1051365 and PRJNA1051380, respectively.

Research involving human participants, their data, or biological material

Policy information about studies with human participants or human data. See also policy information about sex, gender (identity/presentation), and sexual orientation and race, ethnicity and racism.

Reporting on sex and gender	N/A
Reporting on race, ethnicity, or other socially relevant groupings	N/A
Population characteristics	N/A
Recruitment	N/A
Ethics oversight	N/A

Note that full information on the approval of the study protocol must also be provided in the manuscript.

Field-specific reporting

Please select the one below that is the best fit for your research. If you are not sure, read the appropriate sections before making your selection.

☒ Life sciences ☐ Behavioural & social sciences ☐ Ecological, evolutionary & environmental sciences

For a reference copy of the document with all sections, see nature.com/documents/nr-reporting-summary-flat.pdf

Life sciences study design

All studies must disclose on these points even when the disclosure is negative.

Sample size	The exact number of fish used in the study is noted in the main text, figures or figure or figure legend and methods. The exact number of samples used for SCFA analyses is indicated in the methods and figure legends. No statistical method was used to predetermine the sample size.
Data exclusions	No data was excluded.
Replication	Biological replicates for analyses are indicated in the methods and figure legends.
Randomization	Before initiation of the fish feeding trial, salmon were randomized into control and treatment groups to generate equal mean weight in each tank.
Blinding	For the fish trials, blinding was applied to diets. In addition, analyses were run in a treatment-blinding manner.

Reporting for specific materials, systems and methods

We require information from authors about some types of materials, experimental systems and methods used in many studies. Here, indicate whether each material, system or method listed is relevant to your study. If you are not sure if a list item applies to your research, read the appropriate section before selecting a response.

Materials & experimental systems

n/a	Involved in the study
☒	☐ Antibodies
☒	☐ Eukaryotic cell lines
☒	☐ Palaeontology and archaeology
☐	☒ Animals and other organisms
☒	☐ Clinical data
☒	☐ Dual use research of concern
☒	☐ Plants

Methods

n/a	Involved in the study
☒	☐ ChIP-seq
☒	☐ Flow cytometry
☒	☐ MRI-based neuroimaging

Animals and other research organisms

Policy information about studies involving animals; ARRIVE guidelines recommended for reporting animal research, and Sex and Gender in Research

Laboratory animals	For both in vivo trials, pre-smolt salmon (approx 30 gr). For the low dose mannan trial, samples where obtained from the growing salmons (males and females) aged 30 weeks (T0), 35 weeks (T1), 40 weeks (T2) and 45 weeks (T3). For the for the high dose mannan trial, samples were obtained from male and female salmons aged 45 weeks.
Wild animals	No wild animals was used in this study.
Reporting on sex	Both female and male salmon was used in both trials.
Field-collected samples	The fish used in the study conducted at NMBU were reared in recirculated freshwater tanks (14.4±0.4 °C) and kept under continuous light. The fish used in the study conducted at EWOS Innovation were reared in recirculated freshwater tanks (12.0±0.4 °C) and kept under continuous light. Prior to gut content collection, fish were euthanized by using an overdose of tricaine methane sulfonate (MS-222).
Ethics oversight	The experiments were conducted according to the guidelines and protocols approved by the European Union (EU Council 86/609; D.L. 27.01.1992, no. 116) and by the National Guidelines for Animal Care and Welfare published by the Norwegian Ministry of Education and Research. No field collected samples were used in the study.

Note that full information on the approval of the study protocol must also be provided in the manuscript.

Plants

Seed stocks	N/A
Novel plant genotypes	N/A
Authentication	N/A
